# Novel Peptide–Drug Conjugates with Dual Anticancer Activity

**DOI:** 10.3390/ijms252212411

**Published:** 2024-11-19

**Authors:** Siobhán O’Flaherty, Olga A. Luzina, Nadezhda S. Dyrkheeva, Ysaline Krier, Jérôme Leprince, Alexandra L. Zakharenko, Mikhail A. Pokrovsky, Andrey G. Pokrovsky, Olga I. Lavrik, Nariman F. Salakhutdinov, Mihayl Varbanov, Marc Devocelle, Konstantin P. Volcho

**Affiliations:** 1 Department of Chemistry, RCSI University of Medicine and Health Sciences, 123, St. Stephen’s Green, D02 YN77 Dublin, Ireland; mdevocelle@rcsi.ie; 2 SSPC (Synthesis and Solid State Pharmaceutical Centre) Research Centre, V94 T9PX Limerick, Ireland; 3 Department of Medicinal Chemistry, N.N. Vorozhtsov Novosibirsk Institute of Organic Chemistry, 9 Acad. Lavrentjev Ave., 630090 Novosibirsk, Russia; luzina@nioch.nsc.ru (O.A.L.); anvar@nioch.nsc.ru (N.F.S.); volcho@nioch.nsc.ru (K.P.V.); 4 Institute of Chemical Biology and Fundamental Medicine, SB RAS, 8, Lavrentjev Ave., 630090 Novosibirsk, Russia; dyrkheeva.n.s@gmail.com (N.S.D.); sashaz@niboch.nsc.ru (A.L.Z.); lavrik@niboch.nsc.ru (O.I.L.); 5 Laboratoire Lorraine de Chimie Moléculaire, Université de Lorraine, CNRS, L2CM, 54000 Nancy, France; ysalinekrier@sfr.fr (Y.K.); mihayl.varbanov@univ-lorraine.fr (M.V.); 6 Inserm, Rouen Normandie Université, NorDiC UMR 1239, 76000 Rouen, France; jerome.leprince@univ-rouen.fr; 7 Rouen Normandie Université, HeRacLes UMS 51, PRIMACEN, 76000 Rouen, France; 8 V. Zelman Institute for Medicine and Psychology, Novosibirsk State University, 2 Pirogova str., 630090 Novosibirsk, Russia; miha.pokrovsky@gmail.com (M.A.P.); agpokrovsky@gmail.com (A.G.P.)

**Keywords:** antimicrobial peptides, secondary metabolites, peptide–drug conjugates, usnic acid, anticancer

## Abstract

Cationic antimicrobial peptides (AMPs), also called host defence peptides, have established antimicrobial and anticancer activities. Conjugation of an AMP to a bioactive molecule with complementary activity can address some of the clinical limitations of the peptide candidate. This approach has been particularly applied in antimicrobial applications of AMPs, but it remains relatively less explored in the generation of anticancer candidates. In this study, two usnic acid derivatives, based on hydrazinothiazole and benzylidenefuranone pharmacophore moieties, respectively, were conjugated to L-K6, a lysine/leucine-rich AMP, through a new pyrazole ligation intrinsically driven by the cargo molecule. Both components, the usnic acid derivative and the peptide, are selectively active against cancer cells, by targeting the human DNA repair enzyme tyrosyl-DNA phosphodiesterase 1 (TDP1) and through DNA damage, respectively. The two conjugates, based on a hydrazone linkage, exhibited pleiotropic effects, ranging from reduction in the activity of the parent drugs to their conservation or even enhancement. Notably, the conjugates retained some anti-TDP1 activity and displayed intermediate, or even higher, cytotoxicities against glioblastoma cells, compared to their individual components.

## 1. Introduction

Cancer is recognised by the World Health Organization (WHO) as the second-leading cause of death worldwide, with one in six deaths being a consequence of malignancy [1]. In 2020, an estimated 10 million people died from cancer, and approximately 19.3 million new cases were diagnosed; nonetheless, cancer mortality has been reduced in recent years, given the improvements in available treatments and their accessibility [2]. There is a steady development of new anticancer drugs and forms of chemotherapy to combat the high incidence of cancer. However, with chemotherapy as the main form of treatment, drug resistance complicates the matter significantly [3]. Described as the innate (primary) and/or adaptive (secondary) ability of cancer cells to avoid the effects of drugs through a number of mechanisms, this is a major obstacle in cancer therapy [4]. It is one of the reasons warranting the search for novel anticancer agents and/or treatment modalities.

Small molecules represent the most abundant class of drugs in treating a diverse range of diseases [5]. As chemical compounds of low molecular weight, they possess the ability to modulate biochemical processes in order to diagnose, treat, or prevent diseases through a wide range of mechanisms [6]. In recent years, there has been a shift in research from small molecules such as broad-spectrum cytotoxic drugs to more targeted cytotoxic agents, which are often of high potency and low toxicity [7]. Kinases [8], epigenetic regulatory proteins [9], proteasomes [10], and DNA damage repair enzymes [11] are often targeted as a result. 

**Figure 1:**
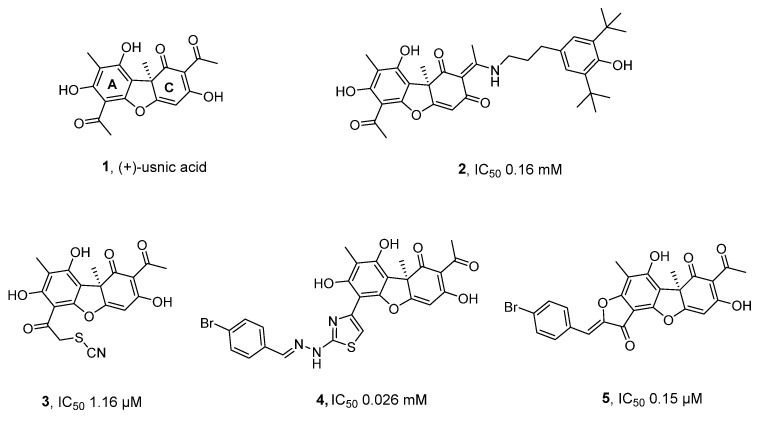
Structures of usnic acid and derivatives with Tyrosyl-DNA-phosphodiesterase 1 (TDP1)-inhibitory activities.

Tyrosyl-DNA-phosphodiesterase 1 (TDP1) is a critical enzyme in the repair of DNA lesions caused by antitumour drugs in the form of topoisomerase 1 (TOP1) poisons such as camptothecin and its derivatives [12]. Thus, the application of TDP1 inhibitors in cancer treatment would increase the toxicity of anticancer therapeutics targeted at topoisomerases [13,14,15]. Some natural products have been identified as promising platforms for developing effective TDP1 inhibitors [16,17,18], such as the secondary metabolite usnic acid (**1**) of lichen origin, with broad biological activity [19]. In recent years, the synthesis of usnic acid derivatives has generated various polycyclic structures with inhibitory activities against TDP1 [20], such as compounds **2** [21], **3** [22], **4** [23], and **5** [19] (Figure 1), the synergistic action of which, when combined with the TOP1 inhibitor topotecan, was confirmed in cell culture experiments and in animal models [23,24]. In particular, two usnic acid derivatives (**4** and **5**), active in the low micromolar–nanomolar range, were identified as promising candidates.

Peptide-based cancer therapy is also among the alternative treatment options that are currently being explored. Malignant cells possess cancer-specific proteins on their membrane, which may be utilised in finding or designing molecules that can selectively bind to these proteins [25]. For example, some peptides specifically recognise and bind to the membrane proteins of tumour cells, with tumoricidal effects. Alternatively, those of a cationic nature are attracted to the anionic phospholipids present on the outer membrane of cancer cells [26,27]. This mechanism is exploited by antimicrobial peptides (AMPs), which are either of natural origin or synthetically produced. Potentially useful against infectious diseases of viral, bacterial, and fungal origins, they have also been shown to possess promising and unique anticancer activities [28]. Structurally, they can be classified as α-helical, β-sheet, extended, or loop peptides, of amphiphatic nature, due to being made up predominantly of hydrophobic and cationic residues [27]. In terms of selectivity, AMPs with anticancer properties are classifiable into two groups: peptides that are active against cancer and microbial cells while not damaging normal mammalian cells, and peptides that are active against all three cell types [28]. However, the mechanism through which anticancer peptides kill malignant cells can be affected by various factors and is not systemically understood in terms of binding, biofunction, and uptake. Among mechanistically characterised AMPs, L-K6 is a leucine/lysine-rich sequence with selective anticancer activity, primarily exerted by DNA binding and nuclear damage [29]. 

Cationic AMPs and the closely related cell-penetrating peptides (CPPs), including L-K6, are able to translocate biological membranes and, therefore, exhibit characteristics of possible delivery vectors for small-molecule drugs, with improved tumour-targeting ability, via potential specific membrane interactions and cell internalisation [30]. By conjugating a small-molecule drug to the peptide, transport of this bioactive cargo into the target cell may be simplified and associated with minimal toxicity, good efficacy, high potency, and the potential to circumvent resistance mechanisms such as efflux pumps. Conversely, some limitations that often affect peptides, such as short half-life and low oral bioavailability [31], can potentially be mitigated through conjugation [32]. This approach has been widely investigated in the modification of AMPs for antimicrobial applications [33,34] but has been comparatively less studied with anticancer candidates [35], particularly in the conjugation of bioactive molecules beyond established chemotherapeutic drugs. 

**Figure 2:**
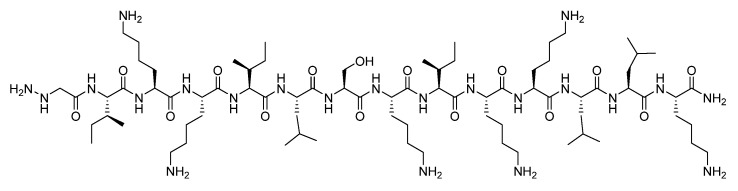
Chemical structure of hydrazinoacetylated L-K6 peptide (**6**).

The work depicted in this paper outlines the preparation of an α-hydrazinoacetylated L-K6 peptide (**6**) (Figure 2) by solid-phase peptide synthesis (SPPS) and its subsequent conjugation with two usnic acid derivatives (**4** and **5**) by a pyrazole ligation, exploiting for the first time an intrinsic structural feature of the latter molecules. These conjugates can mediate the joint and selective (peptide-mediated) delivery to cancer cells of one agent inducing DNA damage and a second agent preventing repair. This combination can broaden the activity of the peptide, particularly against resistant cells, and possibly extend the inhibitory activities of the two components against insensitive cells. Accordingly, the testing of these conjugates, for inhibitory activity against TDP1 and cytotoxicity against human glioblastoma and adenocarcinoma cell lines, is reported here. 

## 2. Results

### 2.1. Synthesis

In addition to being anticancer agents in their own right, the usnic acid derivatives **4** and **5** feature a 1,3-diketone group that can be targeted for effective and selective conjugation to a peptide sequence. It can indeed be exploited in a click chemistry approach by imine-based ligation with an α-hydrazinoacetylated peptide. This approach allows the use of fully deprotected sequences, thereby precluding any restriction in their contents and alleviating concerns related to selectivity (with regard to the conjugation site) or the stability of the cargo under the reaction conditions used for peptide deprotection [36,37]. The usnic acid derivatives **4** and **5** were prepared as previously described in [19,23].

The modified peptide **6** was synthesised using automated SPPS, producing the native sequence with a purity of 95% (Figures S1 and S2). N-terminal modification with a hydrazine linker was performed manually by coupling tri-Boc-α-hydrazinoacetic acid to the resin-bound peptide, followed by standard cleavage and deprotection, yielding **6** with a purity of 93% (Figures S1–S5).

As is known, the reaction of usnic acid with monosubstituted hydrazines proceeds at the C^11^=O carbonyl group with the intermediate formation of hydrazone, and in most cases it is accompanied by intramolecular cyclisation with the formation of a pyrazole ring annelated with a C ring [38]. The only exception is the reacion with arylhydrazines containing strong acceptor substituents, which stops at the stage of hydrazone formation [39]. In all cases, only one isomer of the pyrazole derivative is formed with the arrangement of the five-membered ring at the 2,3 positions of the dibenzofuran framework. This reaction mechanism was exploited here to selectively, efficiently, and stably conjugate the peptide. Practically, while the conjugation reaction is usually carried out in alcohol at room temperature or under heating, here the insufficient solubility of compounds **4** and **5** in anhydrous methanol imposed the use of other solvents. THF, chloroform, and the combination thereof also failed to provide homogeneous solutions, and reactions at a higher temperature (40 °C) remained unsuccessful. Conjugation in anhydrous DMF, at room temperature, was therefore attempted, providing sufficient solubility of both reaction partners and conversion to the desired products, which were subsequently isolated by precipitation from diethyl ether. 

**Scheme 1:**
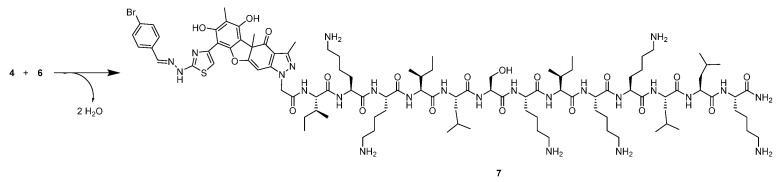
Formation of conjugate **7** from the hydrazinothiazole-based usnic acid derivative **4** and the hydrazinoacetylated peptide **6**.

**Scheme 2:**
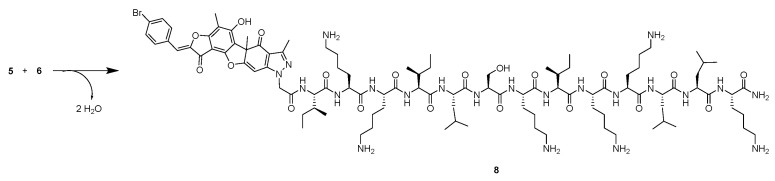
Formation of conjugate **8** from the benzylidenefuranone-based usnic acid derivative **5** and the hydrazinoacetylated peptide **6**.

Mass spectrometry analysis of the peptide conjugate confirmed the loss of two water molecules accompanying the formation of the hydrazone and intramolecular cyclisation akin to the Knorr pyrazole synthesis (Figures S6–S8). Accordingly, conjugation of **5** was performed in the same solvent and manner as **4,** and the same intramolecular cyclisation was observed (Figures S9–S11), with yields of 55% and 63% for conjugates **7** and **8**, respectively (Scheme 1 and Scheme 2, respectively).

### 2.2. TDP1 Inhibition

**Figure 3:**
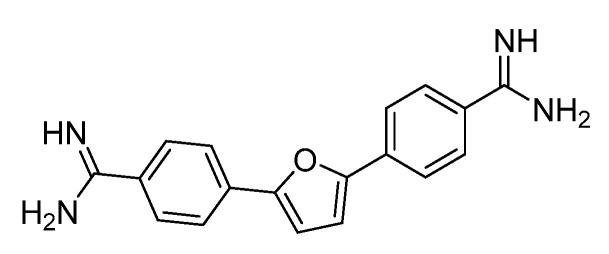
Furamidine (**9**).

These conjugates and their individual components were tested in a real-time fluorescent assay of TDP1 inhibition [40]. Hydrazinoacetylated L-K6 (**6**) was able to inhibit TDP1, but with an IC_50_ value significantly higher than those of its conjugates **7** and **8** (Table 1). The latter showed pronounced inhibitory activity against TDP1, but with approximately 6.5- and 2.3-fold increases in their IC_50s_ compared to their parent TDP1 inhibitors **4** and **5**, respectively. In Table 1, these results are compared to furamidine (Figure 3), the most potent commercial TDP1 inhibitor reported to date, used here as a positive control [41]. 

### 2.3. Cytotoxicity

Next, the conjugates and their parent agents were evaluated in a cytotoxicity assay. Human glioblastoma (SNB19, T98G) and adenocarcinoma (MCF-7) cell lines were used in this study. At a general level, the results showed that the parent TDP1 inhibitors **4** and **5** are more cytotoxic against the adenocarcinoma cell line than against the glioblastoma ones (Table 2). Interestingly, this difference in cytotoxicity is inversed with the conjugates, **7** and **8**, which are generally more cytotoxic against the glioblastoma cell lines than the adenocarcinoma one, while the hydrazinoacetylated peptide itself, like **4** and **5**, is more potent against the latter cells.

Comparing the conjugates and their individual agents, the usnic acid-based candidates (**4** and **5**) are in most cases significantly more cytotoxic than the peptide (**6**) and its conjugates (**7** and **8**). Against the adenocarcinoma cells, the conjugation is either detrimental, with an activity lower than that of both parent compounds, when **4** is the anti-TDP1 candidate, or neutral, with regard to the activity of the less active agent (**6**), when **5** is the anti-TDP1 candidate. Against the T98G glioblastoma cells, the conjugation yields activities that are intermediate between those of the usnic acid and peptide components for both the hydrazinothiazole- and benzylidenefuranone-based candidates (**4** and **5**, respectively). Interestingly, against SNB19 glioblastoma cells, the results compare favourably to those obtained with the adenocarcinoma cells, as here the conjugation either generally maintains the activity of the more active agent (the usnic acid derivative **4**, with conjugate **7**) or even significantly improves the activities of both parent compounds (**5** and **6**, with conjugate **8**). To determine whether some specificity can be achieved against these SNB19 cells, compounds **4**–**8** were also tested against a non-malignant (Vero) cell line. Although only conjugate **7** demonstrated some specificity to SNB19 cancer cells in comparison with Vero cells based on GI_50_ data, for more important GI_80_ data, both conjugates were more toxic to SNB19 cells than to Vero cells.

## 3. Discussion

Peptide–drug conjugates are an emerging class of therapeutic agents. For example, between 1980 and 2018, the proportion of therapeutic peptides entering clinical development as conjugates increased from 5 to 30% [42]. Conjugation of peptides to small molecules can combine the advantages of peptide-based pharmacology with those of traditional medicinal chemistry [32], but the synthesis of such conjugates is associated with a number of challenges. Two main strategies are generally implemented for the conjugation: solid-phase or solution-phase synthesis. The former uses one or more selected residue(s) in an otherwise fully protected sequence. It affords complete selectivity of the conjugation site(s) but requires subsequent cleavage from the resin and global deprotection, under (strongly acidic) conditions that are often not compatible with the stability of the conjugate and/or the non-peptidic component. Alternatively, conjugation in solution with a protected (cleaved) sequence retains selectivity but also faces similar stability limitations, as super acid-sensitive moieties exist as linkers for the solid support but not for all amino acid side-chain protections. A solution-phase conjugation with a fully deprotected peptide circumvents the final deprotection step, but at the expense of selectivity. Otherwise, abiotic functional groups placed on the peptide and the low-molecular-weight entity, as partners of a biorthogonal reaction, can address the latter limitation, but click chemistry [43] can require the use of metal catalysts that might be difficult to remove from the peptide, or the use of reactive moieties (e.g., constrained alkyne) that introduce additional structures significantly deviating from those of the peptide and the conjugated agent. On the other hand, hydrazone ligation, as implemented here, allows for the simple (methodologically and structurally) establishment of a covalent linkage between a peptide modified with a functional group reminiscent of α- or ε-amino groups in peptides and a molecule containing a carbonyl group. Here, the conjugation can be achieved by simply dissolving the macromolecular and molecular components together, does not require any additional reagent or catalyst, is realised with high atom economy, and preserves the integrity of the latter component [44,45,46,47,48].

A peptide–drug conjugate typically comprises three components: the peptide, commonly used as a targeting ligand, a low-molecular-weight agent called cargo, and the linker [49]. The latter can possess important functions with regards to the stability and circulation time of the conjugate, as well as potential release of the drug in its free form at the target site [50]. In the context of cancer therapy, an appropriate linker between the peptide and a cytotoxic drug provides a specific chemical bridge, potentially allowing the peptide to selectively deliver and release (or not) the drug in (the vicinity of) cancer cells [51]. Hence, the choice of linker is determined by the conjugation chemistry and the desired biological outcome. Here, the hydrazine-based linker, introduced by amidation of the peptide’s N-terminus, provides different advantages. Targeting the N-terminus of peptides is indeed a promising strategy to achieve single-site modification at a unique and conserved peptide’s group, which is moreover conveniently solvent exposed for functionalisation by SPPS [52]. The acid-sensitive hydrazone linkage formed is reported to be stable at neutral pH and, therefore, in plasma (pH 7.38–7.42), as well as healthy tissues and cells, but readily cleaved in the acidic conditions found in endosomes and lysosomes, where the pH lies between 4.5 and 6.0 [49,53,54]. Overall, here lies potential for the release of these two agents in cancer cells, if delivered through a lysosomal delivery route [51]. On the other hand, when complete stability of the hydrazone-based conjugate, even at low pH, is desirable, the linker can be reduced to the corresponding hydrazine [55,56]. In our case, enhanced stability is directly provided by the intramolecular (pyrazole) cyclisation, following the hydrazone formation. Indeed, this ligation strategy has already been established with hydrazine peptides and 1,3-diketone groups, having been demonstrated to yield conjugates that are stable at neutral and acidic (4.5) pH for at least seven days at temperatures ≤ 37 °C [57]. The novelty of the present ligation lies in the use of the 1,3-diketone group imbedded in the structure of the bioactive cargos. In addition to this efficient conjugation of molecules containing a 1,3-diketone system, and aside from the independently reported use of C-terminal acyl pyrazoles in native chemical ligation [58], it could be envisaged—owing to the structural similarity of the pyrazole to the well-known triazole, which is itself considered a peptide bond isostere [59]—to extend this effective pyrazole ligation to two peptide fragments, namely, one hydrazine and one 1,3-diketone functionalised sequence.

The mechanism through which anticancer peptides operate in terms of cellular entry is not systemically understood, but through previous studies conducted with L-K6, it is proposed that targeted binding and internalisation of cancer cells are major factors in ultimate cell death [29]. The internalised peptide can cause considerable nucleus damage in MCF-7 cells, without significant disruption of the cytoskeleton and mitochondria. Such results indicate a nucleus-targeting capacity and a potential to serve as a peptidic anticancer drug. N-terminal hydrazinoacetylation of L-K6 does not appear to significantly alter the cytotoxic properties of this peptide (native sequence reported IC_50_ of 23 μM [29]), although slightly lower activities are observed with the modified sequence (GI_50_ of 39 μM). On the other hand, the parent TDP1 inhibitors **4** and **5** possess more pronounced cytotoxic activities in the low micromolar range against all tested cell lines compared to **6**, except for **5** against SNB19 cells, where neither this compound nor the modified peptide **6** demonstrate toxicity in the concentration range tested. Interestingly, in this case, the conjugate **8** has potent cytotoxic activity against these glioblastoma cells (16 μM), while maintaining TDP1 inhibitor activity in the nanomolar range. 

In conclusion, while this conjugation of an AMP and a secondary metabolite did not provide a general approach to produce anticancer candidates with favourable emergent properties, it nonetheless generated a new lead compound against SNB19 cells. The work reported here essentially consisted of a synthetic feasibility study and a primary screening. In addition to a lead identification, other benefits provided by the conjugates presented here (**7** and **8**) include their ease and efficiency of preparation, but also notably their solubility in water, whereas their constitutive compounds **4** and **5** require DMSO for solubility. This is a strong advantage for the potential clinical use of these conjugates in cancer therapy. The next steps in the development of these conjugates would be to study their antitumour efficacy on different cancer cell lines and assess their ability to sensitise topotecan in vitro with different cancer cell cultures, before progressing to in vivo studies [17]. These investigations will comprehensively evaluate the potential of **8** against glioblastoma (and possibly other cancer) cells at a mechanistic level and in terms of selectivity, potency, and capacity to overcome resistance, before being extended subsequently to pharmacokinetic and pharmacodynamic profiling for pre-clinical development. 

## 4. Materials and Methods

### 4.1. Chemistry

Amino acids were purchased from CEM Corporation, Buckingham, UK. All other reagents and solvents were sourced from Sigma-Aldrich (Merck, Dublin, Ireland), unless otherwise stated. Chromatographic analysis and purification by reversed-phase high-performance liquid chromatography (RP-HPLC) was performed on a Shimadzu Prominence HPLC (Mason Technology, Dublin, Ireland) using Phenomenex Gemini columns (5 μm, C18, 110 Å, 4.6 mmD × 250 mmL or 10 mmD × 250 mmL, for the analytical and semi-preparative columns, respectively) (Phenomenex, Macclesfield, UK). The mobile phase, unless otherwise stated, consisted of buffer A: 0.1% trifluoroacetic acid (TFA) in water; and mobile phase B: 0.1% TFA in acetonitrile, with a gradient of 5 to 65% B in 18 column volumes (analytical) or 5 column volumes (semi-preparative), with a flow rate of 1 mL min^−1^ (analytical) or 4 mL min^−1^ (semi-preparative) and wavelength detection between 190 and 800 nm using a PDA detector. Purified peptides were characterised using an Ultraflextreme Matrix-Assisted Laser Desorption Ionisation–Time of Flight (MALDI-TOF) mass spectrometer (Bruker SAS, Wissembourg, France)). Approximately 1 mg of each sample was dissolved in 20 μL of 50% CH_3_CN/0.1% TFA. Then, 1 μL of each sample solution was added to 1 μL of a matrix solution (α-cyano-4-hydroxycinnamic acid, 10 mg in 1 mL of 50% CH_3_CN/0.1% TFA). Routine analysis for reaction monitoring was performed using an Advion Expression Compact Mass Spectrometer (CMS) (Advion, Harlow, UK). Compounds **4** and **5** were prepared according to published procedures [19,23].

#### Peptide Synthesis and Purification

All peptide sequences were assembled by automated SPPS on a CEM Liberty Blue™ Automated Microwave Peptide Synthesiser (CEM Corporation, Buckingham, UK) from 9-fluorenylmethyloxycarbonyl (Fmoc)-protected L-amino acids, according to the Fmoc-*t*Bu strategy with *N*,*N*′-diisopropylcarbodiimide/ethyl cyanohydroxyiminoacetate (Oxyma Pure) coupling chemistry, from a Rink Amide MBHA resin (0.78 mmol/g) on a scale of 0.1–0.25 mM and with final Fmoc deprotection. Single coupling cycles using an excess of Fmoc-amino acids (4 equivalents) were employed unless otherwise stated. The addition of the tri-Boc-α-hydrazinoacetic acid linker was carried out manually using a syringe fitted with a Teflon frit. Removal of Fmoc was carried out using 20% *v*/*v* piperidine in *N*,*N*-dimethylformamide (DMF), and the reaction was monitored by the UV absorbance at 301 nm of the dibenzofulvene–piperidine adduct in the runoff.

After assembly, the peptide was released from the resin by treatment with a cleavage cocktail consisting of 840 μL of trifluoroacetic acid, 100 μL of thioanisole, 50 μL of water, and 10 μL of triisopropylsilane, which was added to the resin and left stirring for 120 min. The peptide was then precipitated with diethyl ether as a white solid, collected by centrifugation, and washed twice with diethyl ether. 

Synthesis of hydrazinoacetylated L-K6 (H_2_NHNCOC)Ile-Lys-Lys-Ile-Leu-Ser-Lys-Ile-Lys-Lys-Leu-Leu-Lys-NH_2_ (**6**): Following washing of the resin-bound (L-K6 native) peptide with CH_2_Cl_2_ and DMF, (Boc)_2_N-N(Boc)CH_2_COOH (195 mg, 0.5 mmol) was coupled using HATU (190 mg, 0.5 mmol) and DIPEA (1.25 mL, 1.0 mmol) activation (120 min at rt with agitation) for each cycle of a double coupling procedure. The resin was then successively washed with DMF and CH_2_Cl_2_ and dried under vacuum. An aliquot cleavage of the peptide from the resin was performed for mass spectrometry analysis. The cleavage, isolation, and purification of the modified peptide were performed as described above. Following a freeze-drying step, the peptide was obtained as a white powder (298 mg, 37%). Analytical HPLC (C18): t_R_ = 28.687 min, 97% purity. ESI MS (*m*/*z*) Calcd for C_77_H_150_N_22_O_15_: 1624.149 Found: 812.6 [M + 2H]^2+^ MALDI-TOF (*m*/*z*) Calcd for C_77_H_150_N_22_O_15_: 1624.149 Found: 1624.202 [M+H]^+^.

Synthesis of L-K6-**4** conjugate (**4**-NHNCOC-Ile-Lys-Lys-Ile-Leu-Ser-Lys-Ile-Lys-Lys-Leu-Leu-Lys-NH_2_, **7**): Peptide **6** (40.7 mg, 0.025 mmol) was added to a solution of **4** (14.6 mg, 0.025 mmol) in anhydrous DMF (1 mL) and left stirring for 24 hours under argon, followed by precipitation using Et_2_O (10 mL), centrifugation, and 2 washes with Et_2_O. The pellet was dissolved in distilled water and lyophilised. Upon drying, a small amount of product was dissolved in water and prepared for mass spectrometry (MALDI-TOF) analysis. Purification of the final peptide was performed by RP-HPLC following standard procedures. Following a freeze-drying step, the peptide was obtained as a pale-yellow powder (30 mg, 55%). Analytical HPLC (C18): t_R_ = 37.593 min, 86% purity. ESI MS (*m*/*z*) Calcd for C_103_H_166_BrN_25_O_19_S: 2170.58 Found: 724.4 [M + 3H]^3+^. MALDI-TOF (*m*/*z*) Calcd for C_103_H_166_BrN_25_O_19_S: 2170.58 Found: 2170.244 [M^+^].

Synthesis of L-K6-**5** conjugate (**5**-NHNCOC-Ile-Lys-Lys-Ile-Leu-Ser-Lys-Ile-Lys-Lys-Leu-Leu-Lys-NH_2_, **8**): This synthesis was performed as described above for **7**. Following a freeze-drying step, the peptide was obtained as a pale-yellow powder (33 mg, 63%). Analytical HPLC (C18): tR = 36.357 min, 94% purity. ESI MS (*m*/*z*) Calcd for C_102_H_163_BrN_22_O_20_: 2095.16 Found: 699.8 [M + 3H]^3+^. MALDI-TOF (*m*/*z*) Calcd for C_102_H_163_BrN_22_O_20_: 2095.16 Found: 2096.349 [M^+^].

### 4.2. Real-Time Detection of TDP1 Activity

The oligonucleotide biosensor 5′-FAM-AAC GTC AGG GTC TTC C- BHQ1-3′, a 16-mer single-stranded oligonucleotide with a 5′-fluorophore (FAM) and a 3′-quencher (BHQ1), was used for real-time fluorescence detection of TDP1 enzyme activity [40]. Recombinant TDP1 protein was expressed in *E. coli* (the pET 16B plasmid containing TDP1 cDNA was kindly provided by Dr. K.W. Caldecott, University of Sussex, United Kingdom) and isolated as described in [60]. The final volume (200 μL) of the reaction mixture contained TDP1 reaction buffer (50 mM Tris-HCl, pH 8.0, 50 mM NaCl, 7 mM β-mercaptoethanol), 50 nM oligonucleotide substrate, and varied concentrations of the tested compounds. Purified TDP1 was added at a final concentration of 1.5 nM. The TDP1 reaction mixtures were incubated at a constant temperature of 26 °C in a POLARstar OPTIMA fluorimeter (BMG LABTECH, Offenburg, Germany). The fluorescence intensity was measured (Ex. 485/Em. 520 nm) every 1 min for 10 minutes. The efficiency of TDP1 inhibition was evaluated by comparing the rate of increase in the fluorescence of the biosensor in the presence of compound **4** or **5** to that of DMSO (1.5%) in control wells. IC_50_ values were determined using an eleven-point concentration response curve by MARS Data Analysis 2.0 (BMG LABTECH), and the slope during the linear phase was calculated. The IC_50_ measurements were carried out in at least three independent experiments.

### 4.3. Cell Culture and Cytotoxicity Assay

Human cancer cells of the SNB19, T98G (cells of human glioblastoma), and MCF-7 (adenocarcinoma) lines, along with cells derived from the kidney of an African green monkey (Vero), were used in this study. These cell lines were obtained from the cell collection of the State Research Center for Virology and Biotechnology “Vector” of Rospotrebnadzor, Koltsovo, Novosibirsk Region. They were cultured in DMEM/F12 medium containing 10% foetal bovine serum, L-glutamine (2 mmol/L), and gentamicin (80 µg/mL) in a CO_2_ incubator at 37 °C. The tested compounds were dissolved in DMSO (**4** and **5**) or H_2_O (**6**, **7**, and **8**) and added to the cellular culture at the required concentrations. Three wells were used for each concentration. The cells that were incubated without the compounds were used as controls. Cells were placed in 96-well microplates and cultivated at 37 °C in 5% CO_2_/95% air for 48 h. The cell viability was assessed through an MTT [3-(4,5-dimethylthiazol-2-yl)-2,5-phenyl-2H-tetrazolium bromide] conversion assay, where 1% MTT was added to each well. Four hours later, the medium was removed, leaving the formazan crystals, and isopropanol was added and mixed for 15 min. The optical density of the samples was measured on a TECAN Sunrise multi-well spectrophotometer at a wavelength of 570 nm, with a reference of 670 nm. The 50% cytotoxic dose (GI_50_) of each compound (i.e., the compound concentration that lowers the amount of cells to 50% in a culture, or decreases the optical density twofold as compared to the control wells) was calculated from the obtained data. Statistical processing of the results was performed using the Microsoft Excel-2007, STATISTICA 6.0, and GraphPad Prism 5.0 programs. The results are given as the average value ± standard deviation from the average. 

## Figures and Tables

**Table 1:** Results of real-time detection of TDP1 activity.

Compounds	IC_50_, nM
**4**	26 ± 11
**5**	150 ± 30
**6**	1800 ± 600
**7**	168 + 23
**8**	340 + 34
**9**	1200 ± 300

**Table 2:** Cytotoxicity study with adenocarcinoma (MCF-7) and human glioblastoma (SNB19, T98G) cell lines.

Cell Line	Cytotoxic Dose ^1^ (µM)	4	5	6	7	8
	GI_50_	2.8 ± 0.07	2.2 ± 0.31	39 ± 6.08	65 ± 15.29	36 ± 1.24
**MCF-7**	GI_80_	4.7 ± 0.21	3.9 ± 0.87	81 ± 16	>100	72 ± 2.4
	GI_90_	5.5 ± 0.41	4.7 ± 1.59	>100	>100	>100
	GI_50_	20.5 ± 2.27	>100	>100	18 ± 0.35	16.5 ± 0.35
**SNB19**	GI_80_	61 ± 1.78	>100	>100	68 ± 3.34	56 ± 0.7
	GI_90_	83 ± 1.87	>100	>100	>100	85 ± 2.32
	GI_50_	10.1 ± 0.67	21 ± 2.6	>100	42 ± 3.02	38 ± 1.22
**T98G**	GI_80_	51 ± 1.08	>100	>100	82 ± 1.78	80 ± 2.86
	GI_90_	90 ± 3.94	>100	>100	>100	>100
	GI_50_	4.1 ± 1.66	>100	64 ± 13.71	33 ± 3.53	18 ± 3.18
**Vero**	GI_80_	48 ± 16.76	>100	>100	>100	>100
	GI_90_	>100	>100	>100	>100	>100

^1^ Cytotoxic dose (GI_50_) is the concentration that lowers the amount of cells to 50% in a culture.

## Data Availability

Data is contained within the article and Supplementary Materials.

## Supplementary Materials

The following supporting information can be downloaded at: https://www.mdpi.com/article/10.3390/ijms252212411/s1.
