# Supplementary material for: Novel Peptide–Drug Conjugates with Dual Anticancer Activity
Source: Int J Mol Sci. 2024 Nov 19;25(22):12411. doi: 10.3390/ijms252212411 (PMC11594562; doi:10.3390/ijms252212411)
Supplement: Supplementary file 1 [file ijms-25-12411-s001.zip › ijms-3291174-supplementary.pdf]

Supplementary Materials

Novel peptide-drug conjugates with dual anticancer activity

Siobhán O'Flaherty, Olga A. Luzina, Nadezhda. S. Dyrkheeva, Ysaline Krier, Jérôme Leprince, Alexandra L. Zakharenko, Mikhail A. Pokrovsky, Andrey G. Pokrovsky, Olga I. Lavrik, Nariman F. Salakhutdinov, Mihayl Varbanov, Marc Devocelle and Konstantin P. Volcho

ESI-MS and RP-HPLC for L-K6 peptide amide.

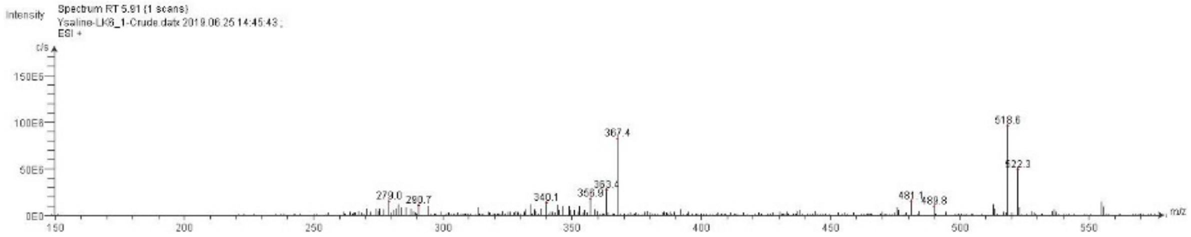

Figure S1 ESI-MS (m/z): Calcd for $C_{75}H_{146}N_{20}O_{14}$ 1551.13, Found: 518.6 $[M+3H]^{3+}$.

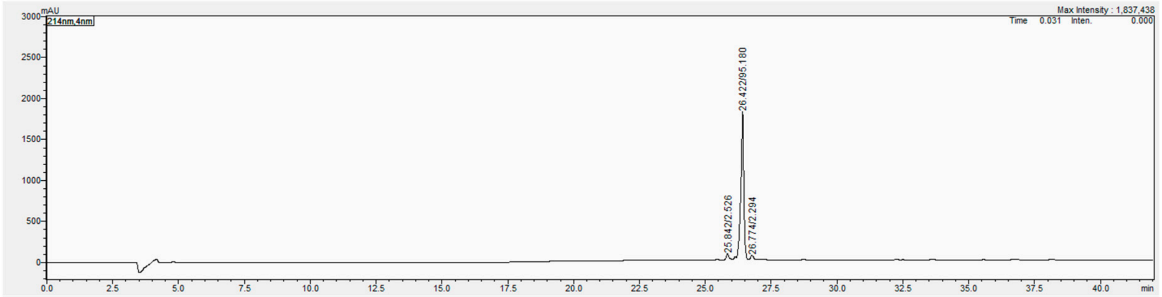

Figure S2 RP-HPLC: t_R = 26.422 min, 95% purity.

ESI-MS, RP-HPLC, and MALDI-TOF MS of hydrazinoacetylated L-K6 peptide, **6**

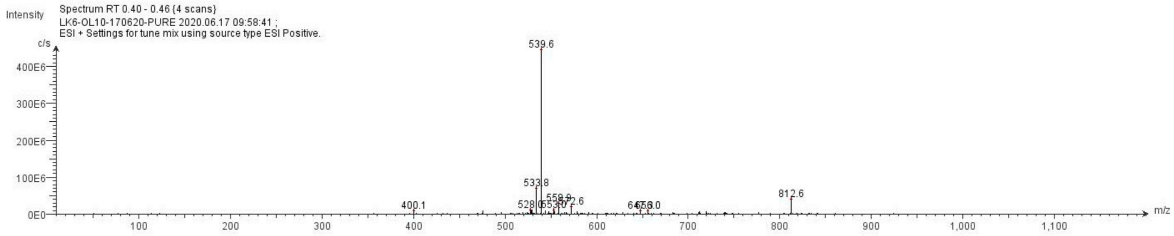

Figure S3 ESI-MS (m/z): Calcd for $C_{77}H_{150}N_{22}O_{15}$ 1624.15, Found: 812.6 $[M+2H]^{2+}$.

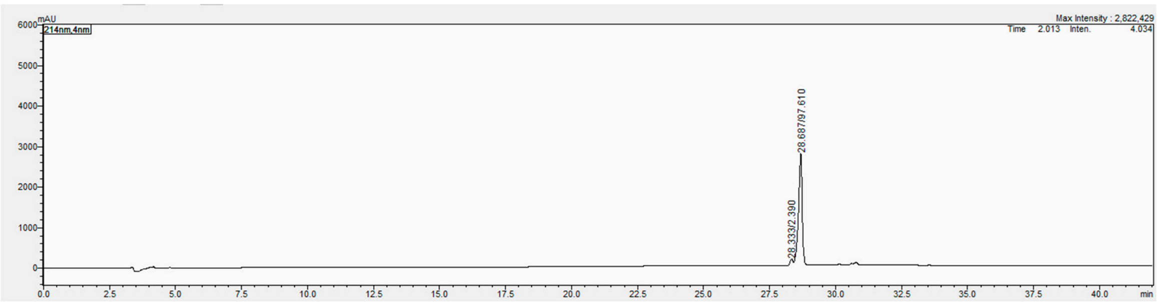

Figure S4 RP-HPLC: t_R = 28.687 min, 97% purity.

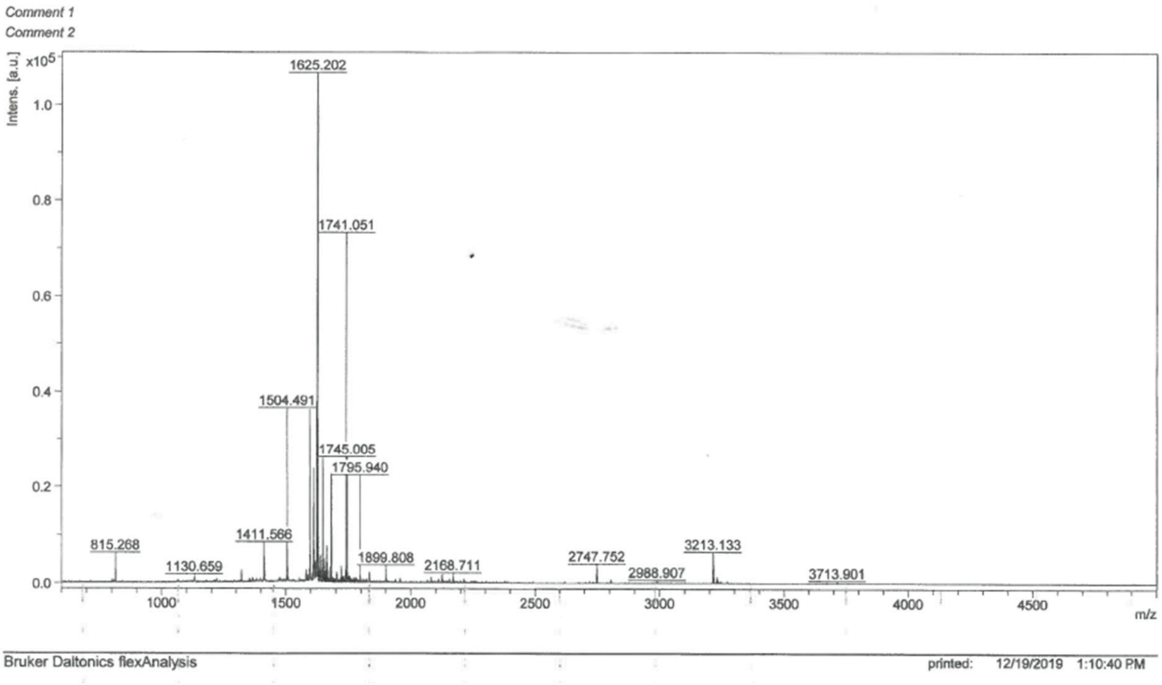

Figure S5 MALDI-TOF MS (m/z): Calcd for $C_{77}H_{150}N_{22}O_{15}$ 1624.15, Found: 1625.202 $[M+H]^{+}$.

ESI-MS, RP-HPLC, and MALDI-TOF MS for the conjugate **7** from the hydrazinothiazole-based usnic acid derivative **4** and the hydrazinoacetylated peptide **6.**

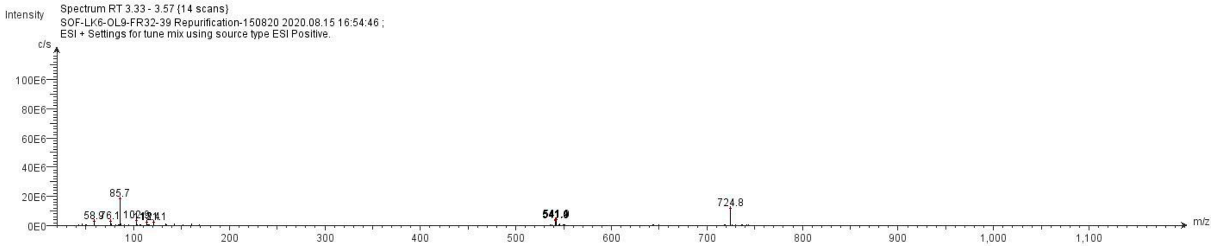

Figure S6 ESI-MS (m/z): Calcd for $C_{103}H_{166}BrN_{25}O_{19}S$ 2170.58, Found: 724.4 $[M+3H]^{3+}$.

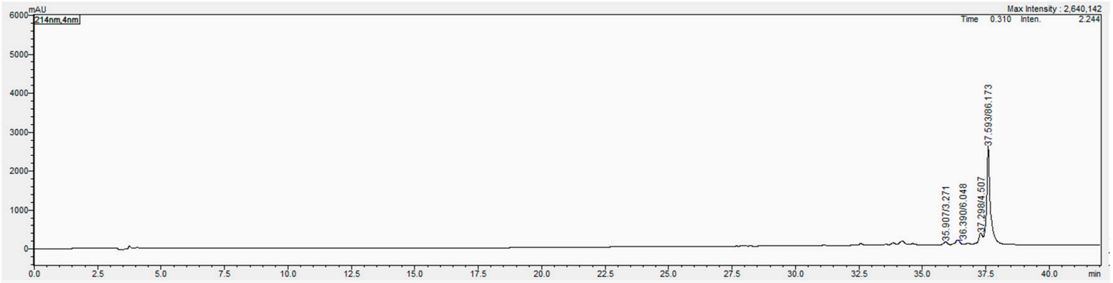

Figure S7 RP-HPLC: t_R = 37.593 min, 86% purity.

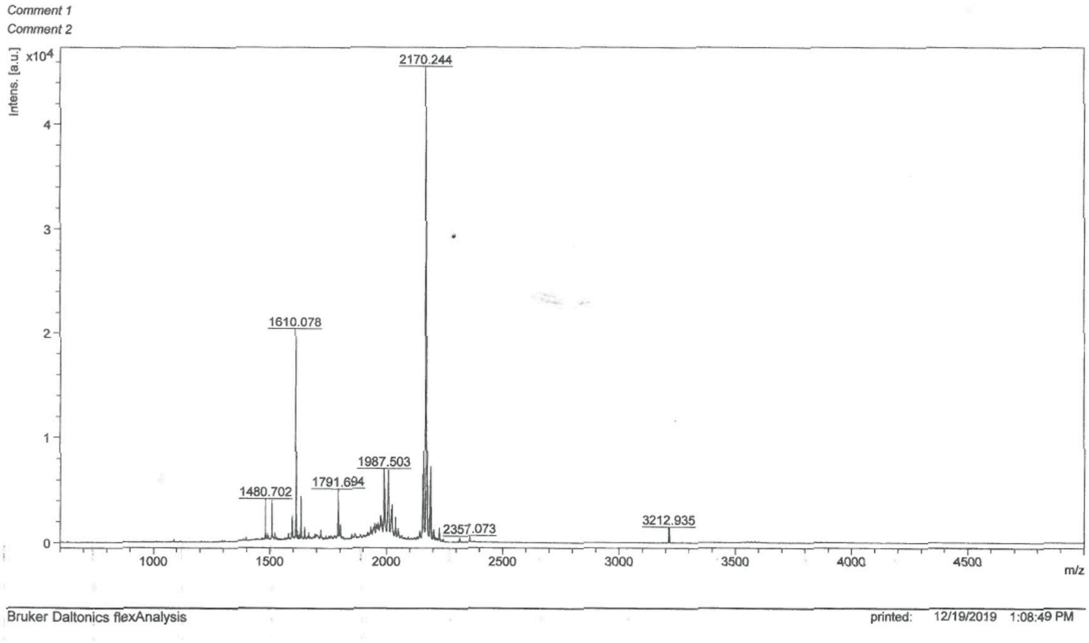

Figure S8 MALDI-TOF MS (m/z): Calcd for $C_{103}H_{166}BrN_{25}O_{19}S$: 2170.58, Found: 2170.244 $[M^+]$.

ESI-MS, RP-HPLC, and MALDI-TOF MS for conjugate **8** from the benzylidenefuranone-based usnic acid derivative **5** and the hydrazinoacetylated peptide **6.**

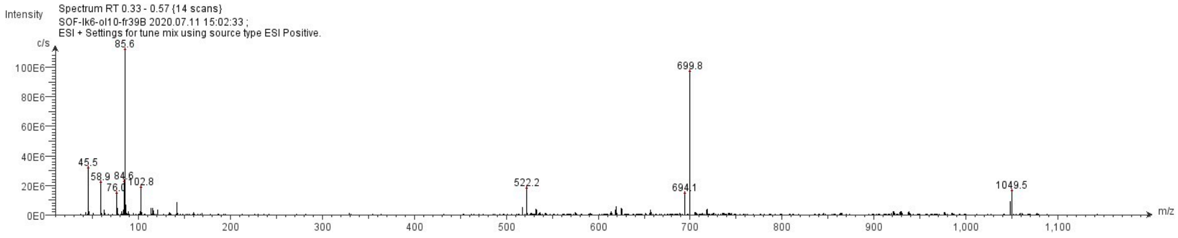

Figure S9 ESI-MS (m/z): Calcd for $C_{102}H_{163}BrN_{22}O_{20}$ 2095.16, Found: 699.8 $[M+3H]^{3+}$, 1049.5 $[M+2H]^{2+}$.

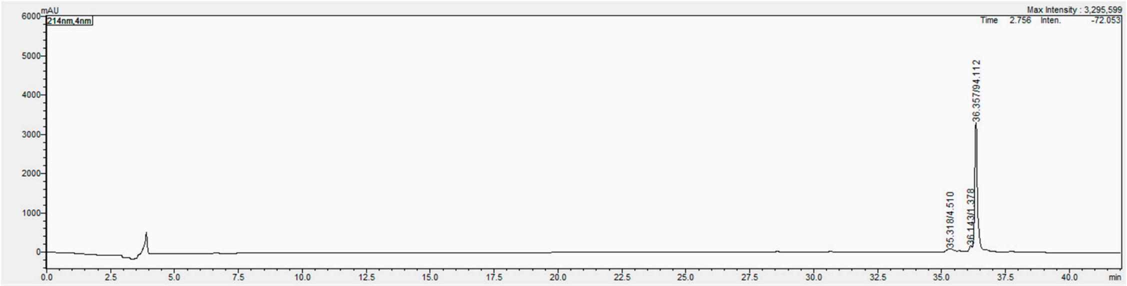

Figure S10 RP-HPLC: t_R = 36.357 min, 94% purity.

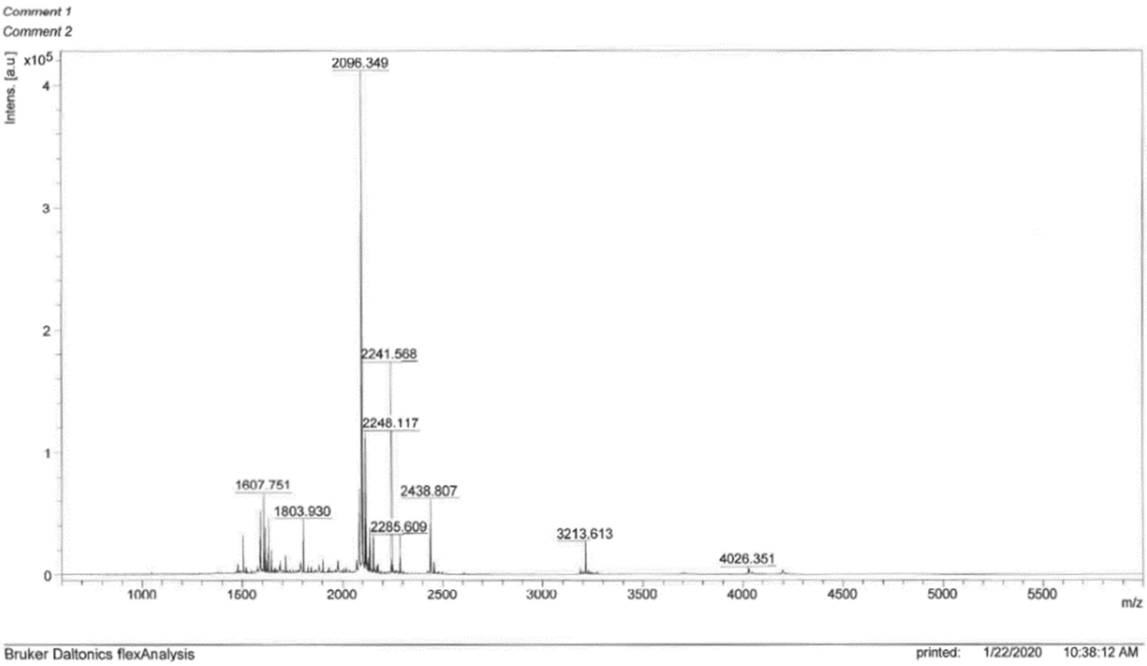

Figure S11 MALDI-TOF MS (m/z): Calcd for $C_{102}H_{163}BrN_{22}O_{20}$ 2095.16, Found: 2096.349 $[M+H]^+$.
